# Metropolitan Society of General Practitioners

**Published:** 1830-10-01

**Authors:** 


### METROPOLITAN SOCIETY OF GENERAL PRACTITIONERS.

OUR readers have seen in our last number that the nucleus of an association has been formed, among a large and very influential class of medical society—the GENERAL PRACTITIONERS, for their mutual and individual interests—for the increase of their own respectability—for the attainment of beneficial laws—for the periodical assemblage of the members—for the cultivation of social intercourse—and for the establishment of a benevolent fund applicable to charitable purposes.

It would be difficult to imagine what possible objection could be started against the formation of a society, having such laudable purposes in view. Yet not only have objections been started, but the self-elected patron of the general practitioners, is using the influence of a partial and unprincipled press, to quash the association altogether! And for what reason? For the *avowed* reason that the title of "GENERAL PRACTITIONER" is a degrading title—and consequently that the society is a degrading society! Such is the *ostensible* objection—the *real* one being mortification at not being admitted a member, and not being able to convert the association into a political engine of discord in the profession. How Mr. Wakley, who was a general practitioner himself, and, whose only elevation since that time, has been into that of general libeller, can make it out that the term is degrading, we know not. HIPPOCRATES and CELSUS, among the antients, were "GENERAL PRACTITIONERS"—and Morgagni, among a host of eminent moderns, was the same. It is absolutely preposterous to attempt to gull the medical public by such a ridiculous charge against a society, when it is well known that Mister Wakley tried all in his power to wriggle himself and his hopeful partner, Mr. Lambert, into the said society, but without success—hinc illæ lacrymæ!

But the name of a society, like the title of a book, will never damn it if it be good, nor save it from dissolution, if bad. If the objects which the Society has held forth be *bonâ fide* objects, they need not fear success—and indeed we are confident that they are sincere in their professions as well as honorable in their intentions. There is one object not stated in the prospectus, but which we have reason to think is contemplated by the Society—the possession of a house, or chambers, where a library and reading room may be established—and where, as in a club, the members may meet and have refreshments or dinners at a very moderate rate. This would be a great benefit and accommodation. Indeed, we are astonished that such an extensive profession, as the medical, has not yet established a club—an establishment which is so easily formed, and so certain of success. The clubs, in fact, are the most flourishing and productive speculations that have been projected in modern times. There is not the smallest doubt but that they will multiply annually. Now the GENERAL PRACTITIONERS have it easily in their power to effect the formation of a club, where they can meet to transact business, as well as to associate around the festive board —and that at very economical expense. We wish them every success, and they may rest assured that the scurrilous or rather JUDAS-LIKE salutations of the reptile press will do them no injury.

